# A semi-automated microscopic image analysis method for scoring Ki-67 nuclear immunostaining

**DOI:** 10.1590/1414-431X2023e12922

**Published:** 2023-11-13

**Authors:** S.M. Fernezlian, C.M. Baldavira, M.L.F. de Souza, C. Farhat, A.F. de Vilhena, J.C.N. Pereira, J.R.M. de Campos, T. Takagaki, M.L. Balancin, A.M. Ab'Saber, V.L. Capelozzi

**Affiliations:** 1 Laboratório de Genômica e Histomorfometria, Departamento de Patologia, Faculdade de Medicina, Universidade de São Paulo, São Paulo, SP, Brasil; 2 Departamento de Cirurgia Torácica, Instituto do Coração, São Paulo, SP, Brasil; 3 Department of General Thoracic Surgery, Georges Pompidou European Hospital, Paris, France; 4 International Perioperative Europrogram, Paris, France; 5 Departamento de Cirurgia Torácica, Hospital Israelita Albert Einstein, São Paulo, SP, Brasil; 6 Divisão de Pneumologia, Instituto do Coração, Faculdade de Medicina, Universidade de São Paulo, São Paulo, SP, Brasil

**Keywords:** Pulmonary typical carcinoid tumor, Chromatin gradient, Cell cycle, Image analysis, Prognosis

## Abstract

Nuclear proliferation marker MIB-1 (Ki-67) immunohistochemistry (IHC) is used to examine tumor cell proliferation. However, the diagnostic or prognostic value of the Ki-67 nuclear staining intensity and location, defined as nuclear gradient (NG), has not been assessed. This study examined the potential association between Ki-67 NG and cell cycle phases and its effect on the prognosis of pulmonary typical carcinoid (PTC) tumors. We propose a method for classifying the NG of Ki-67 during the cell cycle and compare the results between PTC, pulmonary adenocarcinoma (PAD), and breast ductal carcinoma (BDC). A literature review and objective analysis of IHC-stained paraffin sections were used to determine the Ki-67 labeling index and composed a stratification of the NG into NG1, NG2, and NG3/4 categories. A semi-automated image analysis protocol was established to determine the Ki-67 NG in PTC, PAD, and BDC. High intraobserver consistency and moderate interobserver agreement were achieved in the determination of Ki-67 NG in tumor specimens. NG1 and NG2 were lower in PTC than in PAD and BDC. Cox multivariate analysis of PTC after adjusting for age and number of metastatic lymph nodes showed that Ki-67 NG1 and NG2 significantly predicted clinical outcomes. The semi-automated method for quantification of Ki-67 nuclear immunostaining proposed in this study could become a valuable diagnostic and prognostic tool in PTC.

## Introduction

Immunohistochemical staining (IHC) of the nuclear proliferation marker MIB-1 (Ki-67) is extensively used to evaluate tumor cell proliferation and growth (1). Ki-67 is a non-histone DNA-binding protein, and its encoding gene MKI-67 (10q26.2) is upregulated during all cell cycle phases (G1, S, G2, and mitosis) (2). This provides pathologists with a complementary tool to grade tumors in addition to morphologic parameters. The Ki-67 proliferation index (PI), which is determined by immunohistochemical staining, has prognostic and predictive value in several cancers including pulmonary neuroendocrine tumors (3), pulmonary adenocarcinoma (PAD) (4), and breast ductal carcinoma (BDC) (5). However, estimating the Ki-67 PI in a reproducible manner is challenging (5,6). According to the 2021 WHO classification, the Ki-67 PI for pulmonary neuroendocrine neoplasms was established as <5% in typical carcinoids and >20% for atypical carcinoids (7). Furthermore, the role of Ki-67 PI in prognosis prediction is controversial. Some studies demonstrated the prognostic value of the Ki-67 PI in multivariate analysis (8,9), whereas other studies did not support its value (10,11). Therefore, additional methods to evaluate the prognostic role of the Ki-67 PI and to decrease interobserver variation are urgently needed.

Technological advances have improved the estimation of Ki-67 PI evaluation into a fast and quantitative methodology, but with limitations (12,13). Other methods, such as the eyeball method, show high interobserver variation compared with digital counting, whereas manual counting of 2000 cells provides similar results to digital counting (14). There is currently no consensus regarding scoring methods (e.g., hotspots *vs* overall average, digital *vs* manual counting) or even the definition of a Ki-67-positive cell. In addition, the currently available methods are time-consuming and provide only quantitative information without considering the morphological aspects (15).

During routine IHC for the determination of Ki-67 expression, we identified typical and atypical mitoses without difficulty. However, non-mitotic positive nuclei are usually analyzed without considering the differences in the Ki-67 nuclear staining intensity and location, defined as nuclear gradient (NG), which can be clearly visualized. Neoplastic cells contain different Ki-67 immunophenotypes including dot, spot, isolated, or grouped granules, strong and homogeneous karyocytosis, and chromosomes in mitosis (16,17). We recently used the histochemical gradient method for matricellular components and semi-automated quantification to construct an algorithm that facilitates data collection on tumor heterogeneity and histologic classifications, thus providing information on the mechanisms underlying invasion and disease progression (18). Based on previous research, we considered the following issues: 1) whether differences in Ki-67 immunophenotypes according to NG have biological importance; 2) whether these differences are associated with cell cycle phases; 3) whether the nuclei with different staining gradients can be estimated using only the anti-Ki-67 antibody; and 4) whether these differences, compared to Ki-67 PI, affect the prognosis of pulmonary typical carcinoid (PTC) tumors.

This study was designed to develop a stratification system for the Ki-67 nuclear immunostaining gradient based on previous scientific evidence of Ki-67 expression during the cell cycle. In addition, we aimed to develop guidelines for the use of this system in tumors with different proliferative activities. IHC analysis of tissue microarrays was used to estimate the NG of Ki-67 protein expression in PTC, PAD, and BDC.

## Material and Methods

### Exploratory cohort

For more than three decades, investigators have used antibodies to visualize protein abundance, distribution, and localization; variations in the distribution and concentration of Ki-67 are detected as intensely stained nucleoli and full staining of nuclei (16-25). Recent studies have provided information on the role of Ki-67 during the cell cycle; these studies assessed the morphological, functional, and biochemical aspects of Ki-67, as well as its distribution and density during the cell cycle including interphase, chromatin arrangements, and nucleolar shape in relation to Ki-67 (26-34).

After an extensive review of the literature, we created a practical protocol to evaluate and quantify the immunopositivity patterns of Ki-67 during different cell cycle phases in tumor specimens from two cohorts of patients and determine the impact of these patterns on progression-free disease.

### Patient cohorts and samples

The study was approved by the internal ethics committees of the participating institutions under protocol 0572/10, and the need for informed consent was waived because of the retrospective nature of the study. The study included two cohorts of patients as follows:

#### Discovery Cohort

Samples from 99 patients who underwent surgical resection for PTC were obtained from archived formalin-fixed paraffin-embedded (FFPE) histologic sections. The samples were collected at the Clinical Hospital of the University of São Paulo Medical School and at the Hôpital Européen Georges Pompidou from the University of Paris V, where patients underwent surgical resection between January 1, 1981 and December 31, 2016. None of the patients received neoadjuvant therapy. The histologic diagnosis was reviewed according to the 2021 WHO by two lung pathologists (V.L.C. and A.M.A.) (7).

#### Validation Cohort

Tumors with different proliferative activities were included in the study to validate the discovery cohort. This cohort included tumor specimens from 41 patients with PAD and 46 patients with BDC from the Clinical Hospital of the University of São Paulo Medical School.

Demographic data and the clinicopathological characteristics of the patients were obtained from medical records and included age, sex, tumor size, and follow-up information regarding progression-free disease (PFD). Considering that the cohort included cases from as early as 1981, tumor stage was updated according to the 8th Edition of The American Joint Committee on Cancer (AJCC) Cancer Staging and the 8th Edition International Association for the Study of Lung Cancer (IASLC) (19,35).

Table 1 summarizes the clinicopathological and epidemiologic data of the patients.

**Table 1:** Clinicopathologic characteristics of the three cohorts.

	PTC	PAD	BDC	P value
Age (median)^#^				<0.01
<46 years	32.8% (59/180)	2.8% (5/180)	13.3% (24/180)	
≥46 years	21.1% (38/180)	18.9% (34/180)	20% (20/180)	
Gender^#^				<0.01
Male	27.5% (50/182)	21.1% (22/182)	0.0% (0.0)	
Female	25.8% (47/182)	9.3% (17/182)	25.3% (46/182)	
Pathologic stage^†#^				<0.01
I	40.8% (73/179)	8.4% (15/179)	8.4% (15/179)	
II	6.7% (12/179)	7.8% (14/179)	9.5% (17/179)	
IIIA	6.7% (12/179)	5.6% (10/179)	6.1% (11/179)	
Lymph node status^#^				0.03
Negative	45.1% (79/175)	11.4% (20/175)	16.6% (29/175)	
Positive				
>1	4.6% (8/175)	4.0% (7/175)	6.3% (3/175)	
>2	4.6% (8/175)	4.6% (8/175)	1.7 % (3/175)	
>4	1.2% (2/175)	0.0% (0/0)	0.0% (0/0)	
Vital status^#^				<0.01
Dead	7.2% (13/181)	14.4% (26/181)	0.0% (0/0)	
Alive	45.9% (83/181)	7.2% (13/181)	25.4% (46/181)	
Progression-free survival (months)^#^				<0.01
<65	24.6% (32/130)	24.6% (32/130)	100%	
≥65	49.2% (64/130)	1.5% (2/130)	100%	

Person's chi-squared and Fisher's exact tests. PAD: pulmonary adenocarcinoma; BDC: breast ductal carcinoma; PTC: pulmonary typical carcinoid tumor. ^†^According to the 8th edition of The American Joint Committee on Cancer (AJCC) Cancer Staging and the 8th edition of the International Association for the Study of Lung Cancer (19). ^#^Some cases had missing information: age [6]; gender [4]; pathologic stage [7]; lymph node status [11]; vital status [5]; progression free survival [56].

### Technical procedure

#### Tissue microarrays

After histologic review of the original slides, tissue microarrays (TMAs) were constructed from FFPE representative tumor cylinders. Briefly, each tumor cylinder selected based on hematoxylin-eosin (HE) staining was placed in the receiver block according to a previously prepared map, with a 0.3-mm space between samples. For each case, three cylinders were generated in duplicate in the receiver block to avoid sampling bias resulting from physical losses and/or the representativeness of the TMA technology. Then, the TMA blocks were cut into serial 3-µm-thick slices using a manual microtome (Leica Instruments, Germany). Each block produced 70 sections, which were distributed on a marked slide embedded in paraffin and stored in a dark box at -20°C to preserve the antigenicity of the samples.

#### Characterization of nuclear measurements and chromatin

Tumor nuclear measurements were obtained by digital morphometry, and the chromatin intensity was evaluated in HE-stained TMAs. Tumor nuclear morphometry was characterized by measuring each nucleus including nuclear area, nuclear length, nuclear width, and nuclear width/length ratio.

#### Ki-67 detection

After the construction of TMAs, paraffin blocks were cut into 3-μm sections and placed on silane-coated slides. The slides were subjected to IHC for Ki-67 detection (clone MIB-1, Dako, USA; 1:400, pH 6.0). Because the tumor blocks were from 1981 and age can interfere with antigen retrieval and IHC, normalization to other markers (PCNA and cyclin-1) was performed in paired blocks to verify the differences in staining and scoring, and to ensure that the effect on prognosis was not due to quality differences. Procedures and reagents used included Novolink Max Polymer (Novocastra, UK), pressure cooking antigen retrieval, biotinylated rabbit anti‐mouse immunoglobulin G (Dako; dilution 1:400), streptavidin combined *in vitro* with biotinylated horseradish peroxidase (Dako; dilution 1:1000), diaminobenzidine tetrahydrochloride, and counterstaining with Harris hematoxylin (Merck, Germany). Appropriate positive and negative controls were included for each IHC run.

#### Categorization of nuclear gradient

**Figure 1:**
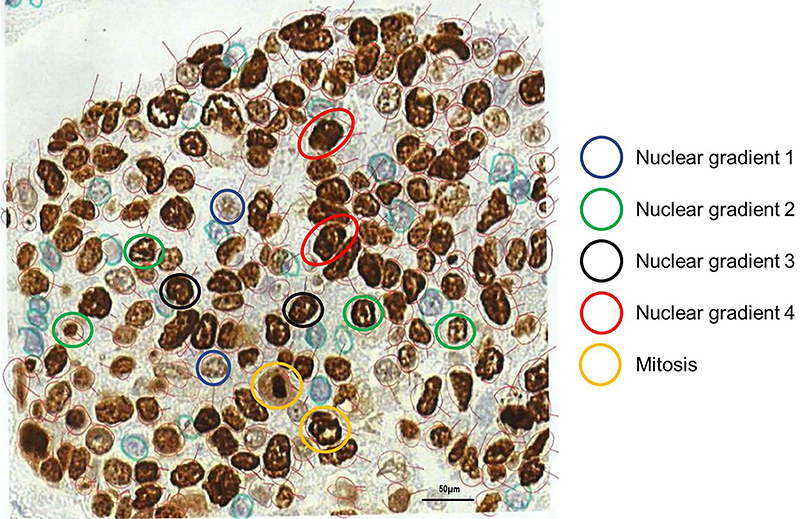
Procedure for the selection and evaluation of nuclei according to the classification criteria. Blue for nuclei gradient 1 (NG1), green for NG2, black for NG3, red for NG4, and yellow for mitoses. Scale bar 50 μm.

Ki-67 grading patterns were classified according to nuclear morphometry, chromatin staining intensity, and location patterns of Ki-67 immunostaining as shown in Figure 1. NG1 included normal-sized nuclei with clear nucleoplasm and without nucleoli, randomly distributed Ki-67 immunopositivity detected as fine or coarse granules, and nuclear membrane Ki-67 positivity. NG2 was characterized by normal-sized nuclei with a clear nucleoplasm and nucleoli, well defined by Ki-67 immunostaining in one or two nodes coinciding with the perinucleolar area, and nuclear membrane positivity for Ki-67. NG pattern 3 (NG3/NG4) was characterized by increased nuclear volume and variable tumor anisocariosis, a nucleoplasm with intense and homogeneous Ki-67 immunostaining, and detectable nucleoli and nodes. Mitotic nuclei (M) were identified by an empty central area and the presence of random peripheral Ki-67 immunostained granules/nodules. In the other mitotic nuclei (metaphase, anaphase, and telophase), Ki-67 immunostaining was detected exclusively on the chromosome.

The stratification of nuclear Ki-67 staining into four patterns was used to develop a protocol through computational semi-automated analysis.

#### Computational semi-automated image analysis protocol

**Figure 2:**
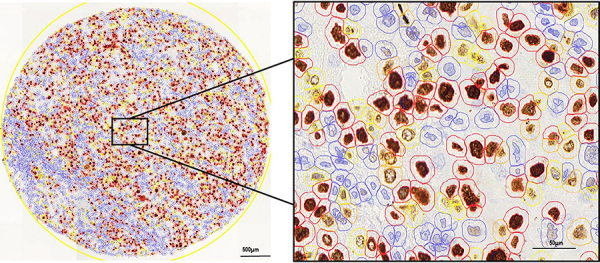
Illustration of microscopic pathology features of Ki-67 immunohistochemistry-nuclear gradient compared with the gold standard method. Scale bars: left image 500 μm and right image 50 μm.

Brightfield slides were scanned using the Panoramic 250 whole slide scanner (3DHistech, Hungary) at ×40 magnification. Stained TMA sections were analyzed using QuPath version 0.2.0‐m4 (Centre for Cancer Research and Cell Biology, University of Edinburgh), an open‐source image analysis platform (36). All cores were evaluated during the scoring process to manually exclude invalid cores (less than 10% of tumor per core or artifacts). For TMA quantification on QuPath, a simple, automated, semi‐assisted method was used considering morphometry and chromatin texture. The desired threshold for NG was selected for the Ki-67 protein after several tests to establish an action flow to convert the manual analysis into a semi-automated analysis. Staining vectors were automatically analyzed for each scanned TMA slide, followed by total tissue area detection, separation of tumor areas from non‐tumor areas in each core, and automatic cellular detection. The Ki-67-positive nuclei were randomly selected and outlined with a light red circle, rotating the right and left sides (Figure 2). The exclusion criteria were overlapping nuclei, blurred nuclei, and weak Ki-67 immunopositivity. Finally, 1000 nuclei were selected for each of the three tumor specimens (n=3,000). Each selected nucleus was labeled according to NG as follows (Figure 1): dark blue circles for NG1, green for NG2, black for NG3, and yellow for mitosis (prometaphase, metaphase, anaphase, and telophase). For each group of 1000 nuclei, all colored circles remained on the virtual slide (Figure 2). The intensity and location of nuclear Ki-67, defined as NG, were assigned through the absorbance threshold from selected cells, tested on each core, and after validation by an expert pathologist, applied to the whole array. The final data were reported as Ki-67 NG in pixels and exported from the software. A corresponding script was then generated and executed on all imported TMA slides from the Ki-67 marker; the detection and export steps for all slides were performed automatically. For comparative purposes in PFD analysis, we also determined the Ki-67 PI.

#### Intra- and interobserver variability

To determine intraobserver variability, each nucleus was evaluated twice by the same observer (M.L.B.). For the interobserver evaluation, the images containing 1000 randomly selected nuclei were organized using PowerPoint software (Microsoft, USA), distributed in 50 slides, and printed for manual counting by two observers (S.F.S. and M.L.F.S.).

### Statistical analysis

Statistical analyses were performed using SPSS software v.18.0 (IBM, USA). Spearman correlation coefficient was used to assess intraobserver consistency using the following categorization for correlations: 0-0.30=very weak; 0.31-0.50=weak; 0.51-0.70=moderate; 0.71-0.90=strong; and 0.91-1.00=very strong (37). Correlations between tumor specimens and NG stratification were analyzed using the mean percentage obtained from the analyses performed for each field. For this analysis, two-way ANOVA with the Bonferroni test for multiple comparisons was used. Relationships between quantitative variables and groups of patients were evaluated by Pearson's chi-squared and Fisher's exact tests. To determine the risk of PFD, the variables were examined by univariate and multivariate analyses using the Cox proportional hazards ratio. A P value <0.05 was considered significant.

## Results

### Categorization of Ki-67 nuclear gradient by morphometry and cell cycle phases

Morphometric analysis was performed in the discovery cohort for PTC and compared with the validation cohort (PAD and BDC). PTCs had a smaller mean nuclear area compared to PAD and BDC (24.38±8.35 µm^2^ *vs* 49.66±3.40 µm^2^ *vs* 33.36±8.41 µm^2^, respectively) and a smaller mean nuclear length than PAD (8.30±0.81 µm *vs* 14.65±2.96 µm, respectively), and nuclei had a more elongated shape in PTC than in PAD and BDC (0.74±0.15 µm *vs* 0.58±0.08 µm *vs* 0.44±0.005 µm). BDC showed smaller width and length than PAD (7.43±0.29 µm *vs* 14.65±2.96 µm and 5.46±0.13 µm *vs* 7.69±2.29 µm, respectively). The mean Ki-67 proliferation index was 0.40% in PTC, 4.31% in PAD, and 3.42% in BDC.

**Figure 3:**
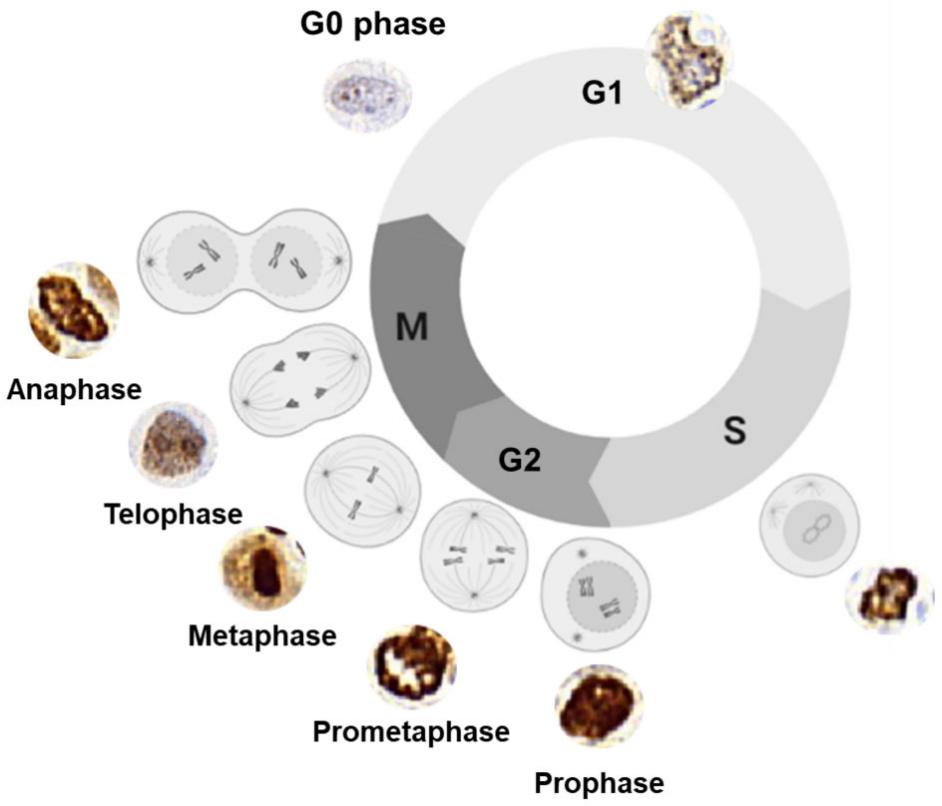
Illustration of the gold standard method of Ki-67 nuclear gradient (NG) determination during the cell cycle obtained from published articles. During the G0 phase, the Ki-67 NG is weakly detectable. In early G1 phase, an inconspicuous nucleolus appears, and Ki-67 proteins can be visualized initiating a new cycle (16,17,25,26). The Ki-67 gradient is detected as multiple spots regularly dispersed in the initial G1 phase (18,19,21). During the progression of the G1 phase, these spots merge with each other, forming irregular clones bordering one or two nucleoli as observed in the late G1 phase (19,21,25). In late G1, S, and G2 phases (interphase), the Ki-67 gradient represents the heterochromatin, perinucleolar heterochromatin, and nucleolus (15,16,19). In S and G2 phases, the Ki-67 gradient intensifies, forming asymmetrical dense granules dispersed throughout the nucleoplasm (13,15). In prophase, the location of Ki-67 staining intensity shifts from the perinucleolar regions, nucleolus, and heterochromatin to the perichromosomal layer and fills the entire nucleus (13,16,33). In prometaphase, the nucleoli cannot be identified, and Ki-67 is densely condensed with peripheral chromosomes, which move to the center, characterizing the metaphasic stage (13,16,33). During telophase, the Ki-67 gradient remains elevated to maintain chromosome separation. When the separation of the daughter cells begins in anaphase, the cell cycle is completed (13,16,33).

As shown in Figure 3, the description of Ki-67 throughout the cell cycle phases according to the literature was applicable and coincided with the categorization of Ki-67 NG patterns (Figure 1). This practical approach was objectively supported by computational image analysis as illustrated in Figure 2.

**Figure 4:**
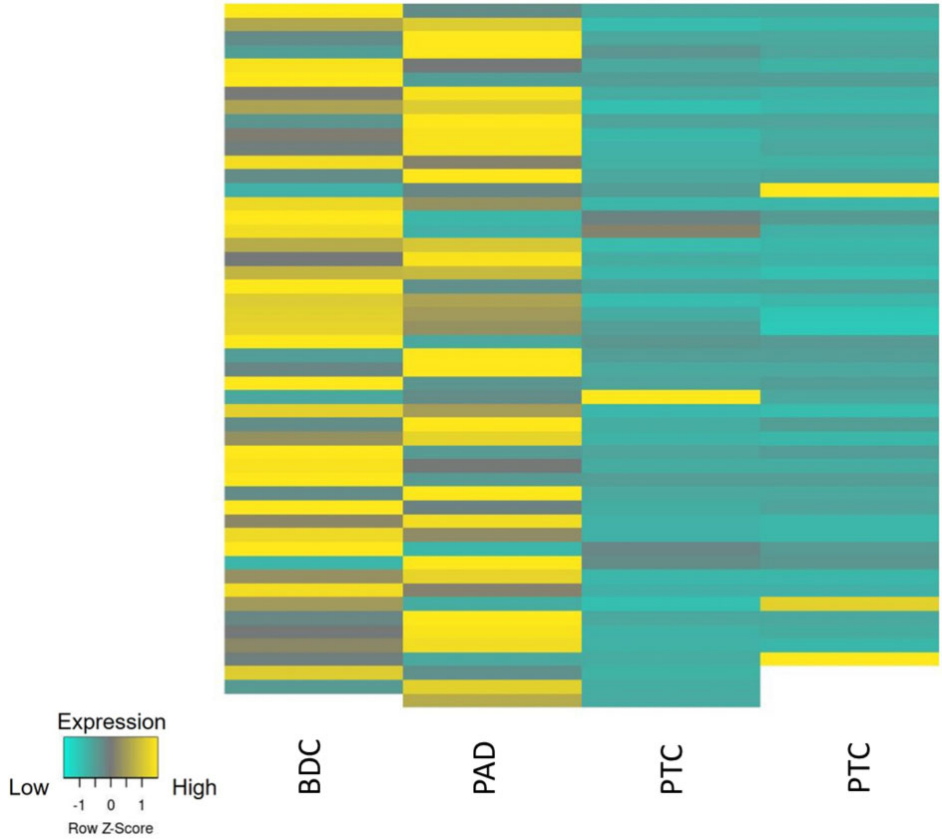
Illustrative heat map for high and low Ki-67 nuclear gradient in breast ductal carcinoma (BDC), pulmonary adenocarcinoma (PAD), and pulmonary typical carcinoid (PTC) tumor.

**Figure 5:**
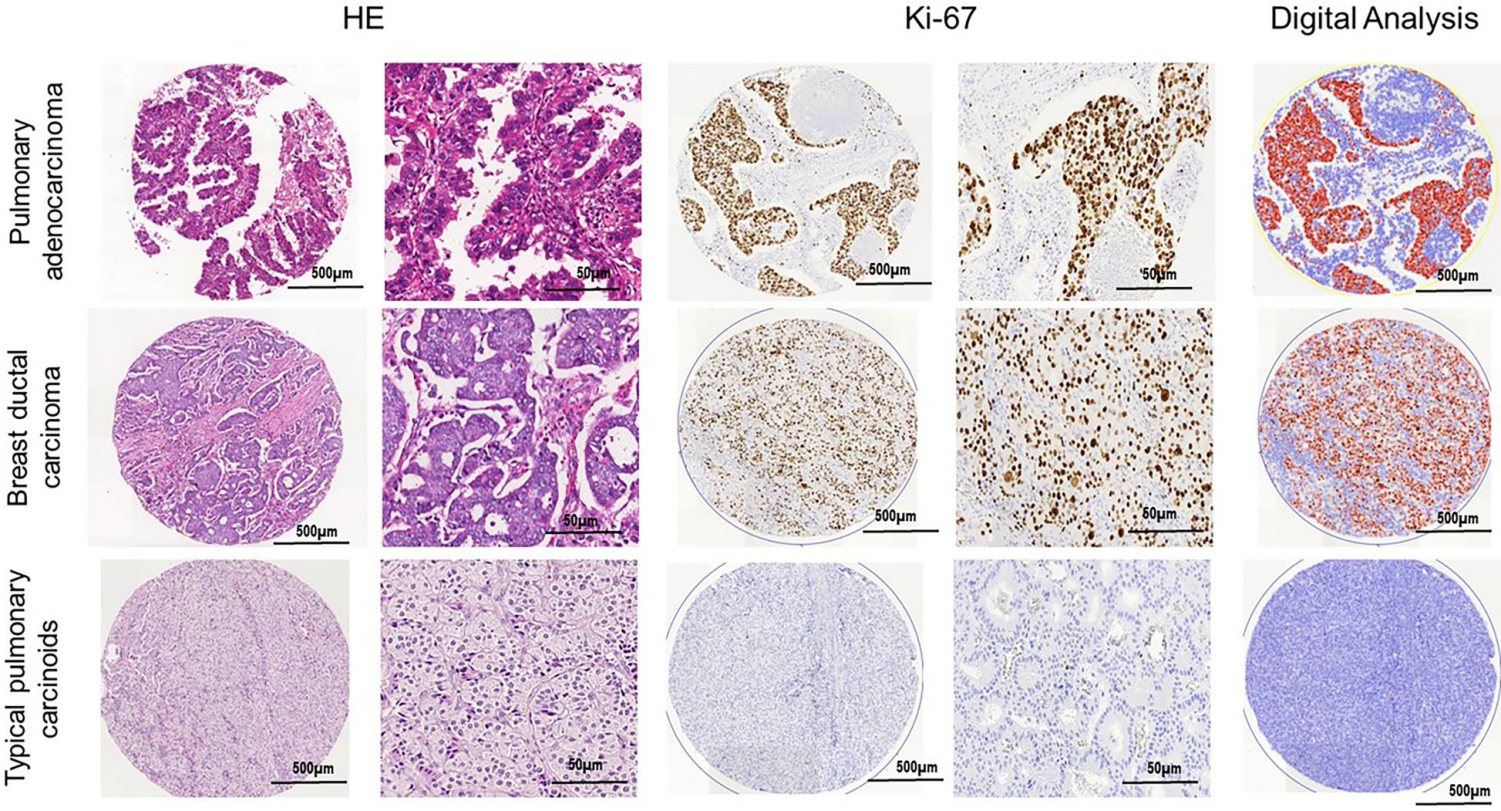
Histological characterization of breast ductal carcinoma, pulmonary adenocarcinoma, and pulmonary typical carcinoids stained by hematoxylin-eosin. Tissue microarray spots stained by Ki-67. Output of the Weka segmentation and of the threshold segmentation for data extraction by coloration. Scale bars, 500 and 50 μm.

The Ki-67 nuclear staining intensity and location, defined as NG, expressed in pixels, in the three tumor groups is summarized in Table 2 and the heat map in Figure 4. Overall, the Ki-67 NG was significantly lower in PTC than in PAD (22.02±6.31 *vs* 351.45±104.37; P<0.001) and in PTC than in BDC (22.02±6.31 *vs* 291.09±84.53; P<0.001). The Ki-67 NG was similar between lung and breast carcinomas (P=0.99). Figure 5 shows the results of HE staining, Ki-67 nuclear chromatin gradient, and digital images of tumor specimens. In PTC, NG2 and NG3 features coincided with a small number of Ki-67-positive nuclei, suggesting that the NG2 and NG3/NG4 patterns are in the same S phase of the cell cycle. The three tumor groups revealed different NGs (Table 2). PTC showed a lower NG1 (16.73±4.78) than PAD (121.60±22.21) and BDC (129.45±23.31), and the differences were statistically significant (P<0.001). NG2 was significantly lower in PTC than in PAD (2.48±0.81 *vs* 60.61±14.84) and lower in PTC than in BDC (2.48±0.31 *vs* 72.93±16.17) (P<0.001). PTC showed a lower NG3/NG4 (2.81±0.72) than PAD (169.24±67.32), and the difference was statistically significant (P=0.001). These results indicated that there was a nuclear chromatin gradient of Ki-67 immunostaining associated with tumor specimens with a predominance of nuclei in NG1 for PTC and NG3/4 for PAD and BDC. Ki-67 nuclear chromatin immunostaining differed between carcinomas and neuroendocrine tumor specimens, suggesting that NG can be effortlessly identified and categorized in different neoplasias in which genetic changes result in cellular heterogeneity. The intraobserver concordance, which was investigated by evaluating 3,000 nuclei in duplicate by the same observer, was very strong: ρ=0.83 for PAD, ρ=0.85 for BDC, and ρ=0.88 for PTC (Spearman's correlation test).

**Table 2:** Comparison of Ki-67 nuclear gradient in pixels according to tumor specimens.

		PAD			BDC		
	n	Mean	SE	n	Mean	SE	Sig
NG1	41	121.60	22.21	46	129.45	23.31	NS
NG2	41	60.61	14.84	46	72.93	16.17	NS
NG3/4	41	169.24	67.32	46	88.71	45.05	NS
		PAD			PTC		
NG1	41	121.60	22.21	99	16.73	4.78	<0.001
NG2	41	60.61	14.84	99	2.48	0.81	<0.001
NG3/4	41	169.24	67.32	99	2.81	0.72	0.001
		BDC			PTC		
NG1	46	129.45	23.31	99	16.73	4.78	<0.001
NG2	46	72.93	16.17	99	2.48	0.81	<0.001
NG3/4	46	88.71	45.05	99	2.81	0.72	NS

PAD: pulmonary adenocarcinoma; BDC: breast ductal carcinoma; PTC: pulmonary typical carcinoid tumor; NG: nuclear gradient; n: number of cases; SE: standard error; Sig: significance; NS: non-significant. ANOVA.

Finally, two observers evaluated the chromatin gradient of Ki-67-positive nuclei by manual counting using printed images from PAD specimens. The results are shown in Table 3 and were compared with the computational count image analysis. A moderate level of correlation was found between manual counting in printed images and digital image analysis for NG1, NG2, and NG3 (P<0.05).

**Table 3:** Nuclear chromatin gradient of Ki-67 by digital image analysis and manual counting in pulmonary adenocarcinoma specimens.

	Digital image analysis *vs* manual counting									
	Digital image analysis			Observer 1			Observer 2			ρ
	n	Mean	SE	n	Mean	SE	n	Mean	SE	
NG1	11/41	9.65	2.48	6/41	19.33	8.43	6/41	15.83	5.04	0.571*
NG2	8/41	64.33	5.91	7/41	125.85	21.01	7/41	99.28	16.63	0.588**
NG3/4	9/41	270.25	32.79	7/41	298.85	26.41	7/41	229.28	22.57	0.421***

NG: nuclear gradient. *P=0.002; **P=0.001; ***P=0.029 (Spearman's correlation).

### Relationship between Ki-67 NG classification and disease stage

Table 4 shows the clinicopathologic characteristics of the study population stratified according to NG. A significant difference was observed between lower NG patterns in PTC compared with those in PAD and BDC (P<0.01). Importantly, tumors in stage IIIA showed higher NG than tumors in stages I and II (P<0.05). Tumors exhibiting higher intensity of NG2 were marginally associated with metastases in more than one lymph node (P=0.09). NG intensity patterns were not correlated with age, gender, tumor size, vital status (alive *vs* dead), and follow-up.

**Table 4:** Clinicopathologic characteristics of the patients according to nuclear chromatin gradient.

	n (%)	Ki-67 NG1	Ki-67 NG2	Ki-67 NG3/4
Age (median)^#^				
<46 years	62 (34.4)	45.29	47.26	46.69
≥46 years	118 (65.6)	51.99	49.85	50.47
Gender^#^				
Female	72 (39.6)	49.18	49.40	49.09
Male	110 (60.4)	47.85	47.63	47.94
Histology				
PAD	41 (22.0)	132.88	133.16	137.79
BDC	46 (24.7)	130.64	136.57	123.96
PTC	99 (53.2)	59.93**	57.07**	61.01**
Tumor size (mean)^#^				
<2.4 cm	6 (13.6)	46.74	47.27	45.08
≥2.4 cm	38 (86.4)	51.94	51.47	53.41
Pathologic stage^†#^				
I	103 (57.5)	48.42	47.14	48.62
II	43 (24.0)	33.71	35.13	32.38
IIIA	33 (18.4)	63.79*	70.04*	63.92*
Lymph node status^#^				
Negative	128 (73.6)	46.36	46.36	47.67
Positive ≥1	46 (26.4)	54.58	57.78***	52.08
Vital status^#^				
Dead	39 (21.5)	48.69	51.88	42.88
Alive	142 (78.5)	47.89	47.38	48.81
Follow-up (median in days)^#^				
<93	81 (62.3)	43.55	47.13	51.28
≥93	49 (37.7)	52.35	48.85	44.79

PAD: pulmonary adenocarcinoma; BDC: breast ductal carcinoma; PTC: pulmonary typical carcinoid tumor; NG: nuclear gradient. *P<0.05; **P<0.001; ***P=0.09 (ANOVA). ^†^According to the 8th edition of The American Joint Committee on Cancer (AJCC) Cancer Staging and the 8th edition of the International Association for the Study of Lung Cancer (19). ^#^Some cases had missing information: age [6]; gender [4]; tumor size [142]; pathologic stage [7]; lymph node status [12]; vital status [5]; follow-up [56].

### Relationship of Ki-67 NG patterns with prognosis

In the entire cohort of 99 patients with PTC, there were 13 deaths caused by occult metastases. In the univariate analysis, the risk of PFD for the entire cohort was significantly affected by age >46 years (P=0.02), female gender (P=0.03), pathological stages II (P=0.04) and IIIA (P<0.001), tumor size >2.0 cm (P=0.009), number of positive lymph nodes ≥1 (P<0.01), NG1 (P=0.03), and NG2 (P=0.04), whereas the Ki-67 PI was borderline significant (Table 5). The robustness of the significant variables identified by Cox univariate analysis was tested in a multivariate analysis, and the resulting model controlled for age (P=0.001) and number of metastatic lymph nodes (P<0.001) was significantly associated with Ki-67 NG1 (P=0.012) and Ki-67 NG2 (P=0.019).

**Table 5:** Variables associated with progression-free disease in 99 pulmonary typical carcinoid patients.

Variables	Univariate analysis^a^			Multivariate analysis^b^	
	HR (95%CI)	B	P value	HR (95%CI)	P value
Age (years, median)					
≤46 (reference)					
>46	1.07 (1.02-1.12)	0.07	0.02	1.22 (1.08-1.38)	0.001
Gender					
Male (reference)					
Female	4.11 (1.12-14.97)	1.41	0.03		
Pathological stage^†^					
I (reference)			<0.001		
II	5.02 (1.01-24.94)	1.61	0.04		
IIIA	22.25 (5.23-94.60)	3.10	<0.001		
Tumor size					
≤2 cm (reference)					
>2 cm	1.04 (1.01-1.08)	0.04	0.009		
Lymph node metastases (number)					
Negative (reference)					
Positive ≥1	0.043 (0.009–0.198)	−3.15	<0.001	0.039 (0.008–0.183)	<0.001
Ki-67 nuclear gradient					
NG1 (mean, 16.69)	1.00 (1.00-1.01)	0.007	0.03	1.01 (1.002-1.020)	0.012
NG2 (mean, 2.48)	1.04 (1.00-1.08)	0.04	0.04	1.07 (1.01-1.14)	0.019
NG3/4 (mean, 2.43) (reference)	1.00 (0.92-1.09)	0.007	0.868		
Proliferation index					
0.39%	1.39 (0.99-1.96)	0.33	0.05		

HR: hazard ratio (β coefficient); CI: confidence interval; B: unstandardized beta (weighted regression); NG: nuclear gradient. The univariate and multivariate analyses employed a Cox proportional hazards model (chi-squared 51.74; P<0.001). ^a^Univariate analysis was carried out without any adjustment in order to generate hazard ratios with confidence intervals for risk factor on survival. ^b^Multivariate analysis was carried out to analyze the effects of several risk factors on survival. ^†^According to the 8th Edition of the International Association for the Study of Lung Cancer (19).

## Discussion

The current study explored differences in Ki-67 immunopositivity based on the Ki-67 nuclear staining intensity and location, defined as gradient, in FFPE tissues and their impact on the prognosis of PTC. We propose a classification system for the gradient of Ki-67-positive nuclei and location during the cell cycle through a comparative analysis of PTC, PAD, and BDC. The results indicated that there was a nuclear chromatin gradient of Ki-67 immunostaining associated with the tumor specimen: NG1 was predominant in PTC, whereas NG2 and NG3/4 were predominant in PAD and BDC. These results suggested that the NG can be easily identified and categorized in different neoplasias in which genetic changes result in cellular heterogeneity. Cox multivariate analysis of PTC after controlling for age and number of metastatic lymph nodes showed that Ki-67 NG1 and NG2 patterns were significantly associated with clinical outcomes, suggesting the predictive value of the differential classification of NG. However, a greater number of tumors, as well as other subtypes of lung neuroendocrine neoplasms, need to be analyzed to confirm these results.

The nuclear protein MIB-1 (Ki-67) is upregulated in the active phases of the cell cycle (28) and is usually assessed by IHC. Previous studies that evaluated Ki-67 as a biomarker focused on identifying the percentage of cells in each cell cycle phase (29,30). Modeling of cell cycle dynamics is being investigated as an important prognostic and predictive biomarker (12,13,28,30). Studies using cultured cells, transmission electron microscopy, and flow cytometry can provide information on the location of Ki-67 during interphase and mitosis (27,31), nucleolus location (32), and Ki-67 in the different cell cycle phases (15,27). However, because these studies were not performed in FFPE tissues, they do not provide pathologists with an ancillary tool to grade tumors or information on morphologic parameters.

The present findings are supported by the literature on Ki-67 biological activity indicating that Ki-67 immunostaining intensity and location, defined as NG, are associated with the cell cycle phases (14,33,38). In this study, PTC was classified as a low NG2 and NG3/4, whereas the high NG1 category may indicate the G1 phase according to progressively decreasing Ki-67 expression. In healthy cells, the cell cycle is precisely regulated and encompasses G1-S-G2-M phases with an inactive/quiescent G0 state. Although cancer cells also progress through four distinct cell cycle phases (39), the control of the different phases may be altered (40). After anaphase, the nuclear protein Ki-67 is degraded evading from the cell cycle in G0. However, if the daughter cells continue in the cycle after division, the fine fragments of Ki-67 from quiescent cancer cells can re-enter the cell cycle and promote new Ki-67 synthesis in G1 (26,34). This may explain why cytotoxic chemotherapy is often ineffective in solid tumors including PTC in G1 phase. By contrast, Ki-67 positivity in PAD and BDC led to the categorization of these tumors as NG2 and NG3/4, topographically suggesting an association with S and G2 phases. This is consistent with the study by Braun et al. (14), who reported that in G2, Ki-67 expression increases substantially and fills the nucleus, as detected by IHC. In addition, Cuylen et al. (27) report that the nuclear protein Ki-67 (encoded by the MKI-67 gene), a constituent of the mitotic chromosome, prevents chromosome assembly, thus favoring individual chromosome motility. This phenomenon could not be understood in cancer cells in the S/G_2_ phase, when they are most sensitive to cytotoxic chemotherapy.

Our results are relevant for the prognosis of patients with PTC. Univariate analysis showed that the risk of progressive disease (HR) for the entire cohort was significantly affected by age >46 years, female gender, pathological stages II and IIIA, tumor size >2.0 cm, number of positive lymph nodes ≥1, and NG1 and NG2. However, when these variables were tested in a multivariate analysis, the resulting model showed that patients older than 46 years with more than one metastatic lymph node and PTC with Ki-67 NG1 or NG2 had higher risk of progressive disease. Ki-67 PI was not a significant factor predicting disease progression.

In summary, the present findings indicated that the semi-automated quantification of the NG for Ki-67 immunostaining may become a potential tool for diagnosis and prognosis prediction in PTC.
